# Galangin Inhibits Cholangiocarcinoma Cell Growth and Metastasis through Downregulation of MicroRNA-21 Expression

**DOI:** 10.1155/2020/5846938

**Published:** 2020-06-11

**Authors:** Yang Zou, Rong Li, Dabin Kuang, Meiling Zuo, Wenqun Li, Wei Tong, Li Jiang, Min Zhou, Yin Chen, Wencheng Gong, Lijuan Liu, Fangfang Tou

**Affiliations:** ^1^Department of Pharmacy, Jiangxi Cancer Hospital, Nanchang, Jiangxi, China; ^2^Pharmaceutical College, Guangxi Medical University, Nanning, Guangxi, China; ^3^The Second Affiliated Hospital of University of South China, Hengyang, Hunan, China; ^4^Office of Good Clinical Practice, Affiliated Changsha Hospital of Hunan Normal University, Changsha, Hunan, China; ^5^Department of Pharmacy, Second Xiangya Hospital, Central South University, Changsha, Hunan, China; ^6^Department of Oncology and Respiratory Medicine, Jiangxi Cancer Hospital, Nanchang, Jiangxi, China

## Abstract

Galangin, a natural flavonoid product derived from the root of galangal, is emerging as a promising anticancer agent against multiple cancers. Yet, whether it also has antitumor effects on cholangiocarcinoma (CCA) and the underlying mechanism is still unknown. Herein, we demonstrate that galangin exhibits multiple antitumor effects on CCA cells including decreases cell viability; inhibits proliferation, migration, and invasion; and induces apoptosis. Moreover, those phenotypic changes are associated with downregulated microRNA-21 (miR-21) expression. To support, overexpression of miR-21 blocks galangin-mediated antisurvival and metastasis effects on CCA cells. Mechanically, galangin increases the expression of phosphatase and tensin homolog (PTEN), a direct target of miR-21, resulting in decreased phosphorylation of AKT, a protein kinase which plays a critical role in controlling survival and apoptosis. In contrast, overexpression of miR-21 abrogates galangin-regulated PTEN expression and AKT phosphorylation. Taken together, these findings indicate that galangin inhibits CCA cell proliferation and metastasis and induces cell apoptosis through a miR-21-dependent manner, and galangin may provide a novel potential therapeutic adjuvant to treat CCA.

## 1. Introduction

Cholangiocarcinoma (CCA), derived from the epithelial cells of either the intrahepatic, perihilar, or extrahepatic bile ducts, is a very poor prognostic malignancy with a 5-year survival rate less than 10% [1, 2]. Results from recent epidemiological observation studies demonstrate that the incidence of CCA is steadily increasing globally in the past decades [3, 4]. Unfortunately, over 70% of CCA patients are diagnosed in an advanced stage and those patients are not eligible for surgical resection or liver transplantation due to the extraordinary invasiveness of CCA [5]. Moreover, accumulating evidence from numerous clinical trials indicate that cisplatin plus gemcitabine therapy, the current standard of care for first-line treatment of advanced CCA, increases the median survival by less than 8-12 months, which is still far from the patient's anticipation [6–8]. Thus, an urgent clinical need exists to develop novel therapeutic agents for CCA treatment.

Accumulating data from clinical and experimental studies demonstrated that microRNAs (miRNAs) are emerging as promising targets for developing novel therapeutic strategies to treat cancers [9]. For example, miR-21 is highly expressed in samples from CCA patients compared with the noncancerous biliary epithelium and the circulating miR-21 levels serve as a potential diagnostic, prognostic biomarker for CCA [10–13]. In a mouse tumor xenograft model, overexpression of miR-21 promotes CCA growth by increasing the tumor size and weight, whereas inhibiting miR-21 suppresses tumor formation [11, 14]. Moreover, downregulation of miR-21 expression promotes multiple CCA cell lines including CAK-1, HuCCT1, TFK-1, KKU-100, and RBE cell apoptosis and inhibits metastasis [14–16], suggesting a key role of miR-21 in CCA cell survival and function. Furthermore, inhibition of miR-21 increases CCA cells sensitivity to gemcitabine therapy [12]. Hence, targeting miR-21 holds great promise as a novel therapeutic strategy for CCA treatment.

Accumulating evidence demonstrate that galangin, a natural flavonoid product extract from the root of galangal, exhibits multiple anticancer effects against various tumors. For instance, galangin inhibits cell growth and metastasis in breast cancer, glioma, and oesophageal and laryngeal carcinoma cells and limits tumor growth in various mouse tumor xenograft models [17–19]. In addition to direct antitumor effects on cancer cells, galangin also attenuates the drug resistance to cisplatin treatment, a widely used anticancer drug in CCA treatment [20]. These data suggest that galangin can be a potential adjuvant for clinic cancer therapy. Yet, whether galangin also has antitumor effects on CCA cells and the underlying mechanism is still unknown. Thus, the aim of the present study is to investigate the effects of galangin on CCA cells and whether the underlying mechanism is through regulating miR-21 expression.

## 2. Materials and Methods

### 2.1. Cell Culture and Transfection

Human intrahepatic CCA cell line HCCC9810 and CCA cell line TFK-1 were purchased from the American Type Culture Collection and cultured in RPMI-1640 (KGM31800, KeyGen Biotech) supplemented with 10% fetal bovine serum (A3161002C, Gibco) and 100 U/ml of penicillin and streptomycin in a moisture incubator at 37°C with 5% CO_2_. Cells were passaged less than five times for all experiments. HCCC9810 and TFK-1 cells were plated on 96-well plates at 5,000/well, 12-well plates at 120,000/well, or 6-well plates at 250,000/well and allowed to grow to 70%-80% confluence and treated with galangin (50, 100, 150, or 200 *μ*M; 282200, Sigma) for 24 hours. For transfection, HCCC9810 and TFK-1 cells were transfected with agomir nonspecific control (NC) (miR4N0000001-4-5, RiboBio), miR-21 agomir (miR40000076-4-5, RiboBio), antagomir nonspecific control (NC) (miR3N0000001-4-5, RiboBio), or miR-21 antagomir (miR30000076-4-5) at 100 nM following the manufacturer's instructions and cells were treated with galangin (150 *μ*M) 12 hours post transfection for 24 hours.

### 2.2. Cell Viability by CCK-8 Assay

HCCC9810 and TFK-1 cells were plated on 96-well plates and treated with galangin (50, 100, 150, or 200 *μ*M) for 24 hours. After treatment, 10 *μ*l CCK-8 solution (CK04, DOJINDO) was added into each well and incubated for 2 hours. The absorbance was measured at 450 nm using an ELISA plate reader (DTX880, Beckman).

### 2.3. Cell Proliferation Assay

Cell proliferation was measured using a commercial EdU assay kit (BeyoClick™ EdU Cell Proliferation Kit with Alexa Fluor 488) (C0071S, Beyotime) according to the manufacturer's instruction. Briefly, HCCC9810 and TFK-1 cells were plated on 96-well plate with or without transfection and treated with galangin as described above. After treatment, the culture medium was switched to fresh RPMI-1640 medium containing 10 *μ*M EdU and incubated for 3 hours. Then, cells were fixed in 4% paraformaldehyde (PFA) for 15 minutes, followed by incubation with 0.3% Triton-X 100 for 15 minutes and then switched to Click buffer for another 30 minutes of incubation under dark condition at 37°C. Images were captured using a fluorescence microscope (AMAFD2000, Thermo Fisher). The number of EdU-positive cells per view was quantified from randomly acquired images.

### 2.4. Cell Migration and Invasion

Cell migration and invasion were determined using transwell assay plate (24-well insert, 8 *μ*M pore size) (3422, Corning). In brief, cells after treatment were washed twice with PBS and resuspended in the serum-free medium at a density of 1 × 10^6^/ml. 200 *μ*l of the suspended cells was added to the insert of the Matrigel-coated (354166, BD Bioscience) membrane. The inserts were placed into the 24-well plates containing 700 *μ*l RPMI1640 supplemented with 10% FBS and incubated for 24 hours at 37°C with 5% CO_2_. The cells in the insert that did not migrate or invade through the pore were carefully removed by scraping with wet cotton swab. The migrated or invaded cells were fixed in 4% PFA for 15 min and stained with 0.1% crystal violet solution (G1062, Solarbio) for 20 min prior to count under a light microscope (Nikon, Japan).

### 2.5. Wound Healing Assay

Wound healing assay was performed by measuring cell migration. Briefly, HCCC9810 cells were plated on 6-well plate with or without transfection and were treated. After cells reached 90% confluence, the linear wound was scratched using a 200 *μ*l pipette tip. Floating and damaged cells were removed by washing with PBS three times. Then, cells were treated with or without galangin. After 48 hours, scratched cells were photographed with an inverted microscope (Nikon, Japan). The migration rate was calculated as follows: migration rate (%) = (scratch distance at 0 h − scratch distance at 48 h)/scratch distance at 0 h × 100.

### 2.6. Cell Apoptosis Analyses by Flow Cytometry (FACS)

Cell apoptosis was assessed using an Annexin V-FITC Apoptosis Detection Kit (KGAV102, KeyGEN BioTech) according to the manufacturer's instruction. In short, after treatment, cells were washed twice with PBS and resuspended in binding buffer. Then, cells were incubated for 5 minutes with Annexin V and PI. FACS was performed on a BD FACSCanto II flow cytometer. Data were analyzed with FlowJo software (TreeStar Inc., Ashland, OR).

### 2.7. RNA Isolation and Real-Time Polymerase Chain Reaction (RT-qPCR)

For RNA isolation, RNAiso Plus reagent (9109, Takara) was used based on the manufacturer's protocol. The commercial Reverse Transcriptase kit (RR036A, Takara) was used for generated cDNA, and RT-qPCR was performed using the TB Green real-time PCR kit (RR420A, Takara). Expression of miR-21-5p levels was normalized to U6. The specific primers for miR-21-5p (MQPS0000835-1-200) and U6 (MQPS0000002-1-100) were purchased from RiboBio. Changes in expression were calculated using a deltadelta Ct method.

### 2.8. Western Blot

Proteins were isolated using RIPA buffer (P0013B, Beyotime) with protease inhibitor (ST505, Beyotime) and phosphatase inhibitor (P1081, Beyotime), and the concentrations were measured using a bicinchoninic acid (BCA) kit (P0012S, Beyotime). Lysates (60 *μ*g/lane) were separated by 12% SDS-PAGE gels and subsequently transferred to PVDF membranes. The membranes were blocked with 5% BSA for 1 hour at room temperature and subsequently incubated with corresponding antibodies as follows: GAPDH (1 : 1000, ab8245, Abcam), T-AKT (1 : 1000, 2920, CST), p-AKT (1 : 1000, 4060, CST), PTEN (1 : 1000, 9188, CST), MMP-9 (1 : 1000, 3852, CST), Vimentin (1 : 1000, 3932, CST), caspase 3 (1 : 1000, 14220, CST), cleaved caspase 3 (1 : 1000, 9664, CST), Bcl-2 (1 : 1000, 60178, Proteintech), or Bax (1 : 1000, 60267, Proteintech) at 4°C overnight. After incubation, the membranes were washed three times with 0.1% TBST and incubated with HRP-conjugated goat anti-mouse (BA1051, Boster) or goat anti-rabbit (BA1055, Boster) secondary antibody for 1 hour at room temperature; the signal was detected using enhanced chemiluminescence reagent (P0018S, Beyotime). The intensity of each band was measured using the Imaging J System (Bio-Rad, USA).

### 2.9. Statistical Analysis

Statistical analysis was performed using GraphPad Prism version 7.0 (GraphPad Software Inc., CA). Unpaired Student's *t*-test was used to determine significant difference between two groups. One-way ANOVA followed by the Brown-Forsythe test and Bartlett's test was used for compare difference in multiple groups. All the data were presented as mean ± SEM. *P* < 0.05 was considered statistically significant.

## 3. Result

### 3.1. Galangin Reduces CCA Cell Viability and Proliferation and Induces Cell Apoptosis

**Figure 1:**
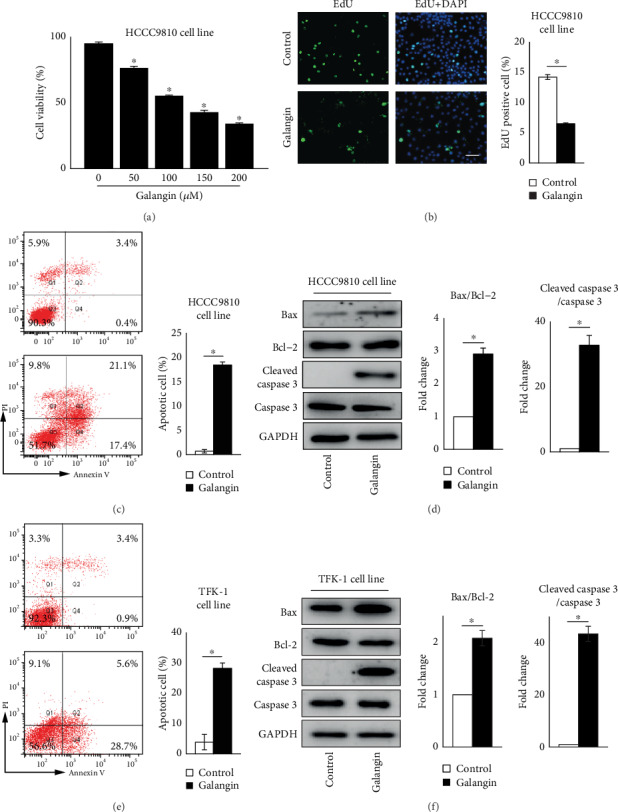
Galangin inhibits cell viability and proliferation and induces apoptosis in cholangiocarcinoma cells. (a) CCK-8 analysis of cell viability in different concentrations of galangin (0, 50, 100, 150, or 200 *μ*M)-treated cholangiocarcinoma (CCA) cell line HCCC9810 cells for 24 hours. *n* = 6 independent experiments. (b) EdU analysis of cell proliferation in galangin (150 *μ*M)-treated HCCC9810 cells for 24 hours. *n* = 5 independent experiments. Scale: 20 *μ*M. FACS analysis of cell apoptosis in galangin (150 *μ*M)-treated HCCC9810 cells (c) or TFK-1 cells (e) for 24 hours. *n* = 3 independent experiments. Western blot analysis of Bax, Bcl-2, cleaved caspase 3, and caspase 3 expression in galangin (150*μ*M)-treated HCCC9810 cells (d) or TFK-1 cells (f) for 24 hours. *n* = 3 independent experiments. Values are given as means ± SEM. ^∗^*P* < 0.05.

To investigate whether galangin also affects CCA cell survival including proliferation and apoptosis, we first treated human intrahepatic CCA cell line HCCC9810 cells with various concentrations of galangin at 50, 100, 150, or 200 *μ*M, respectively, for 24 hours. As shown in Figure 1(a), CCK-8 assay revealed that the cell viability was significantly decreased in galangin-treated HCCC9810 cells in a dose-dependent manner, and galangin at 150 *μ*M reached the 50% inhibitory growth concentration. Thus, galangin at 150 *μ*M was selected for all subsequent experiments. In agreement with the cell viability assay results, cell proliferation examined by EdU assay exhibited 54% reduced EdU-positive cells in galangin-treated HCCC9810 cells compared to those cells treated with vehicle control (Figure 1(b)). Furthermore, FACS assessment of cell apoptosis revealed a 26-fold and 6.3-fold increased apoptotic cells in galangin-treated HCCC9810 cell line and CCA cell line TFK-1 cells, respectively (Figures 1(c) and 1(e)). The galangin-induced CCA cell apoptosis was further confirmed by Western blot analysis as indicated by increased protein expression of cleaved caspase 3 levels and the ratio of Bax to Bcl-2 in both HCCC9810 and TFK-1 cell line (Figures 1(d) and 1(f)). Taken together, these results demonstrate that galangin can reduce CCA cell viability and proliferation and induce cell apoptosis.

### 3.2. Galangin Inhibits CCA Cell Migration and Invasion

**Figure 2:**
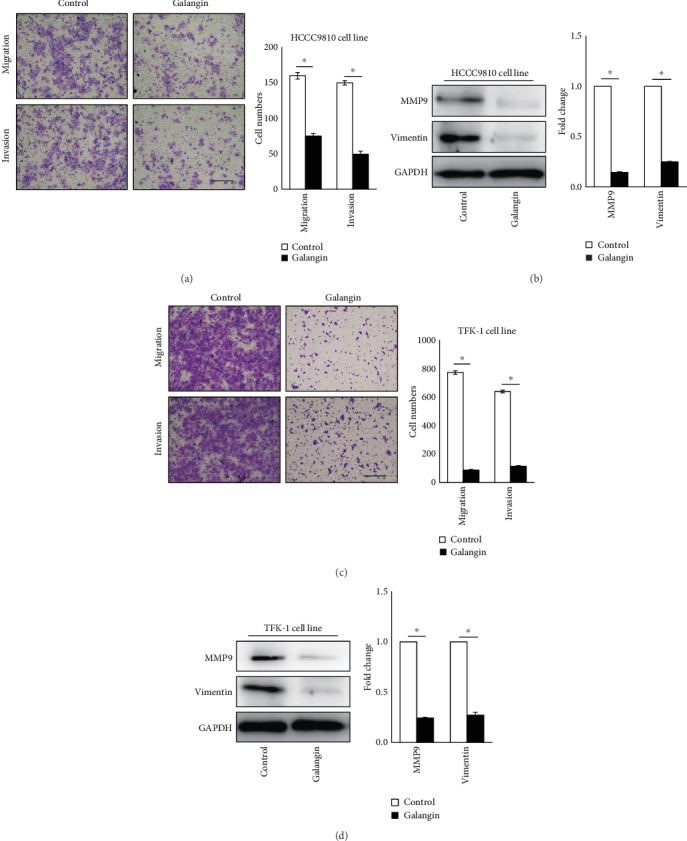
Galangin inhibits migration and invasion in cholangiocarcinoma cells. Matrigel-coated Transwell analysis of migration and invasion in galangin (150 *μ*M)-treated HCCC9810 cells (a) or TFK-1 cells (c) for 24 hours. Scale: 50 *μ*M. *n* = 3 independent experiments. Western blot analysis of MMP9 and Vimentin expression in galangin (150 *μ*M)-treated HCCC9810 cells (b) or TFK-1 cells (d) for 24 hours. *n* = 3 independent experiments. Values are given as means ± SEM. ^∗^*P* < 0.05.

The antimigration and anti-invasion effects of galangin on CCA cells were determined using a Matrigel-coated Transwell method. As shown in Figures 2(a) and 2(c), compared with vehicle control-treated cells, galangin treatment exhibited a significant reduction of migrated and invaded cell numbers by 53% and 67%, respectively, in HCCC9810 cells and by 89% and 82%, respectively, in TFK-1 cells. Matrix metalloproteinase 9 (MMP9) and Vimentin play a crucial role in mediating migration and invasion processes, resulting in accelerated tumor metastasis [21, 22]. In line with Matrigel-coated Transwell assessment, protein levels of MMP9 and Vimentin were dramatically reduced by 85% and 75%, respectively, in HCCC9810 cells (Figure 2(b)), and by 75% and 72%, respectively, in TFK-1 cells (Figure 2(d)). Collectively, these data demonstrate that galangin treatment decreases MMP9 and Vimentin expression and inhibits migration and invasion in CCA cells.

### 3.3. Galangin Reduces CCA Cell Proliferation and Induces Cell Apoptosis in a miR-21-Dependent Manner

**Figure 3:**
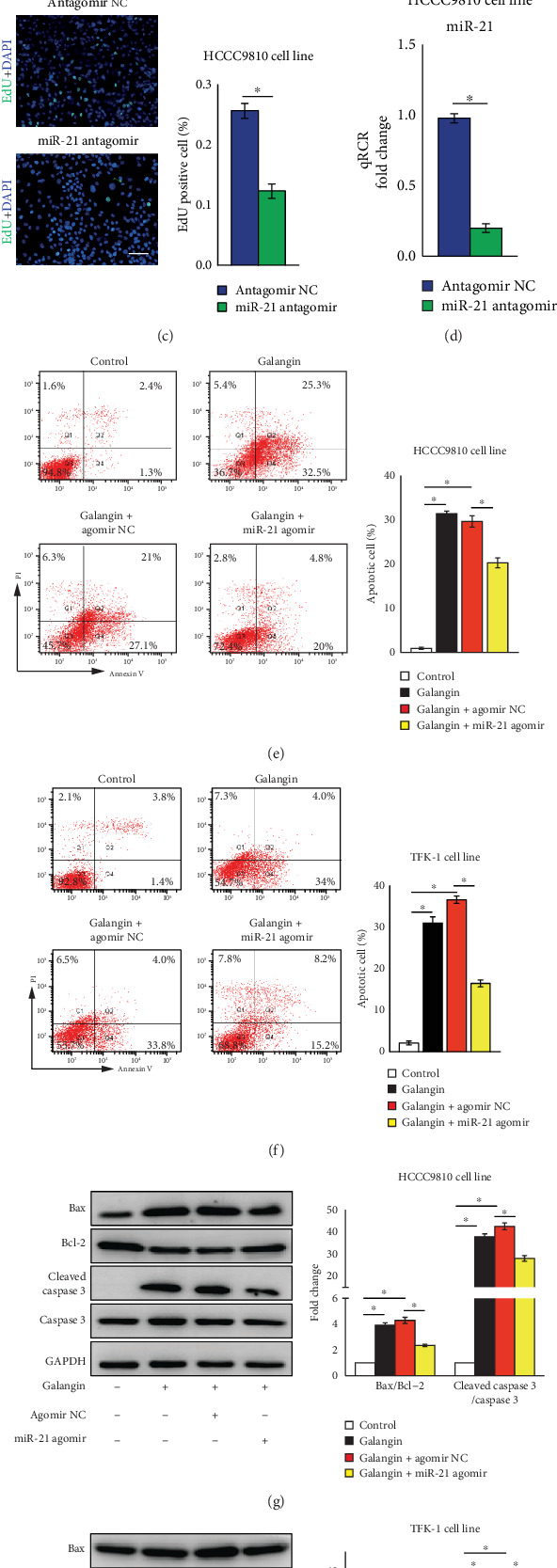
Overexpression of miR-21 abrogates galangin-reduced cell proliferation and galangin-induced cell apoptosis in cholangiocarcinoma cells. HCCC9810 or TFK-1 cells were transfected with 100 nM agomir nonspecific control (NC), miR-21 agomir, antagomir NC, or miR-21 antagomir for 24 hours as indicated elsewhere. (a) HCCC9810 cells were treated with galangin (150*μ*M) for 24 hours and harvested for real-time PCR analysis of miR-21 expression. *n* = 3 independent experiments. (b) HCCC9810 cells were treated with galangin (150*μ*M) for 24 hours and harvested for EdU analysis of cell proliferation. *n* = 5 independent experiments. Scale: 20 *μ*M. (c) HCCC9810 cells were transfected with 100 nM antagomir NC or miR-21 antagomir and harvested for EdU analysis of cell proliferation. *n* = 5 independent experiments. Scale: 20 *μ*M. (d) Real-time PCR analysis of miR-21 expression in antagomir NC or miR-21 antagomir-transfected HCCC9810 cells. *n* = 3 independent experiments. FACS analysis of cell apoptosis in galangin (150 *μ*M)-treated HCCC9810 cells (e) or TFK-1 cells (f) for 24 hours. *n* = 3independent experiments. Western blot analysis of Bax, Bcl-2, cleaved caspase 3 and Caspase 3 expression in galangin (150*μ*M)-treated HCCC9810 cells (g) or TFK-1 cells (h) for 24 hours. *n* = 3 independent experiments. Values are given as means ± SEM. ^∗^*P* < 0.05.

Accumulating evidences suggest that miR-21 is highly expressed in CCA cells and contributes greatly to the pathogenesis of CCA through promoting cell survival and metastasis [10–16]. To investigate whether the antitumor effects of galangin on CCA cells is through fine-tuning miR-21 expression, we first examined miR-21 expression in galangin-treated HCCC9810 cells. Using real-time PCR analysis, the expression of miR-21 was reduced by 45% in galangin-treated CCA cells compared with those cells without galangin treatment (Figure 3(a)). This promoted us to further examine the potential role of miR-21 in galangin-mediated antitumor effects. Cell proliferation analyzed by EdU assessment revealed that overexpression of miR-21 using miR-21 agomir blocked galangin-inhibited CCA cell proliferation compared with those galangin-treated CCA cells transfected with agomir nonspecific control (agomir NC) (Figure 3(b)), while knockdown miR-21 expression using miR-21 antagomir promoted HCCC9810 cell proliferation compared with those antagomir NC-transfected cells (Figure 3(c)). Moreover, cell apoptosis measurement by FACS using Annexin V and PI double staining exhibited that miR-21 overexpression abrogated galangin-induced cell apoptosis in both HCCC9810 and TFK-1 cells (Figures 3(e) and 3(f)). Furthermore, we observed that galangin treatment increased cleaved caspase 3 protein expression and the ratio of Bax to Bcl-2 in both HCCC9810 and TFK-1 cell line was abrogated by miR-21 overexpression (Figures 3(g) and 3(f)). The efficiency of miR-21 overexpression by miR-21 agomir and miR-21 knockdown by miR-21 antagomir was confirmed by real-time PCR as shown in Figures 3(a) and 3(d). Taken together, these findings indicate that the effects of galangin on cell proliferation and apoptosis in both HCCC9810 and TFK-1 cells, at least in part, depend on repressing miR-21 expression.

### 3.4. MiR-21 Mediates Galangin-Induced Inhibitory Effects on CCA Cell Migration and Invasion

**Figure 4:**
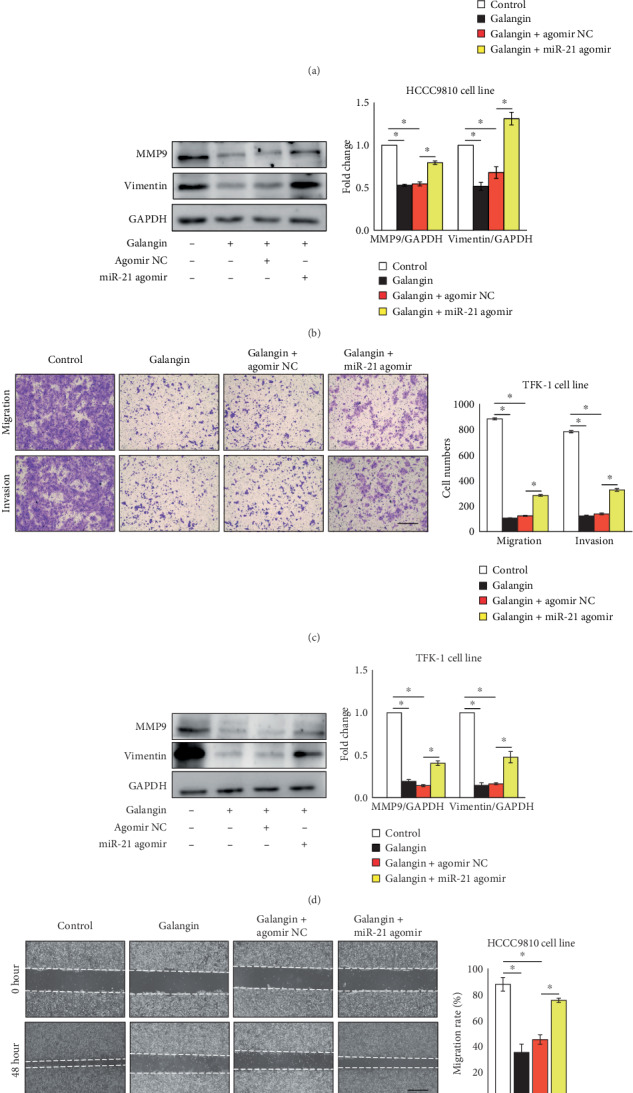
Overexpression of miR-21 abrogates galangin-inhibited cell migration and invasion in cholangiocarcinoma cells. HCCC9810 or TFK-1 cells were transfected with 100 nM agomir nonspecific control (NC) or miR-21 agomir for 24 hours as indicated elsewhere. Matrigel-coated Transwell analysis of migration and invasion in galangin (150 *μ*M)-treated HCCC9810 cells (a) or TFK-1 cells (c) for 24 hours. Scale: 50 *μ*M. *n* = 3 independent experiments. Western blot analysis of MMP9 and Vimentin expression in galangin (150 *μ*M)-treated HCCC9810 cells (b) or TFK-1 cells (d) for 24 hours. *n* = 3 independent experiments. (e) Wound healing assay analysis of migration and invasion in galangin (150 *μ*M)-treated HCCC9810 cells. Scale: 200 *μ*M. *n* = 3 independent experiments. Values are given as means ± SEM. ^∗^*P* < 0.05.

Overexpression of miR-21 abrogates the antiproliferation and proapoptosis effects of galangin on CCA cells (Figure 3). Thus, we next investigate the effects of miR-21 overexpression on galangin-mediated inhibition of cell migration and invasion. As shown in Figures 4(a) and 4(c), compared to galangin-treated CCA cells transfected with agomir NC, overexpression of miR-21 rescued the migratory and invasive ability as indicated by the increased number of migrated and invaded cells in both HCCC9810 and TFK-1 cell treatment with galangin. Consistently, the protein expression of MMP9 and Vimentin in both galangin-treated HCCC9810 and TFK-1 cells transfected miR-21 agomir was significantly decreased compared with that of galangin-treated cells transfected with agomir NC (Figures 4(b) and 4(d)). Moreover, the migration rate was significantly decreased in HCCC9810 cell-transfected miR-21 agomir compared with those galangin-treated cells transfected with agomir NC (Figure 4(e)). Taken together, these data suggest that miR-21 mediates the antimigratory and anti-invasive effects of galangin on CCA cells.

### 3.5. Galangin Inhibiting the PTEN/AKT Pathway Activity Depends on Decreasing miR-21 Expression in CCA Cells

**Figure 5:**
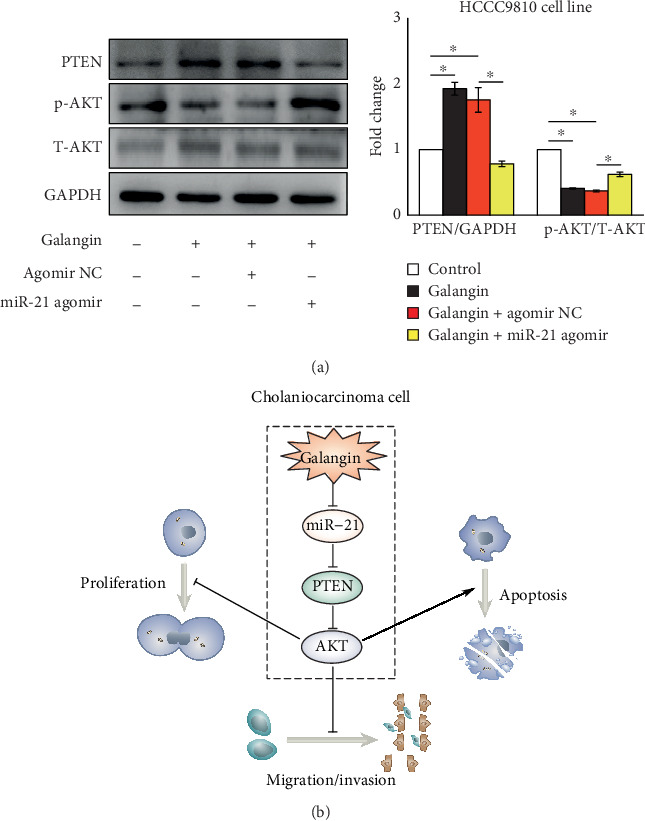
Overexpression of miR-21 increases galangin-reduced PTEN/AKT signaling in cholangiocarcinoma cells. HCCC9810 cells were transfected with 100 nM agomir nonspecific control (NC) or miR-21 agomir for 24 hours. (**a**) Western blot analysis of PTEN expression and phosphorylation of AKT in galangin (150 *μ*M)-treated HCCC9810 cells for 24 hours. *n* = 3 independent experiments. (b) Schema of galangin reduces cholangiocarcinoma cell growth and metastasis. After galangin treatment, it decreases miR-21 expression, led to increased PTEN expression, an effect that reduces phosphorylation of AKT in cholangiocarcinoma cells, resulting in reduced cell growth and metastasis. Values are given as means ± SEM. ^∗^*P* < 0.05.

The PTEN/AKT pathway plays a critical role in controlling cell survival and apoptosis [12, 23]. More importantly, previous studies demonstrated that PTEN is a direct target of miR-21 [12]. Thus, we next examined the effects of galangin on PETN/AKT pathway activity and whether these effects also depended on miR-21 expression. Compared with vehicle control-treated CCA cells, the protein expression of PTEN was increased by 2-fold and the phosphorylation of AKT was reduced by 65%, respectively, in CCA cell treatment with galangin (Figure 5(a)). In contrast, in galangin-treated CCA cells, transfected miR-21 agomir significantly decreased the PTEN protein expression by 33% and increased the phosphorylation of AKT by 2-fold, respectively, compared with those galangin-treated cells transfected with agomir NC (Figure 5(a)). These data suggested that galangin inhibits the PTEN/AKT pathway by decreasing miR-21 expression in CCA cells.

## 4. Discussion

Earlier studies identified that galangin exhibited antitumor effects on multiple cancers, however, no evidence in CCA. In the present study, we demonstrated that galangin inhibits cell proliferation, migration, and invasion and promotes apoptosis in both intrahepatic CCA cell line HCCC9810 and CCA cell line TFK-1 cells (Figures 1 and 2). Moreover, using a complementary approach by overexpression of miR-21, we found that galangin mediated those antitumor effects on CCA cells, at least in part, through downregulation of miR-21 expression (Figures 3 and 4). Finally, we identified that galangin increases PTEN expression, a direct target of miR-21, resulting in decreased AKT activation, while these effects are abrogated by miR-21 overexpression (Figure 5(a)). Taken together, these data indicated that the miR-21-mediated PTEN/AKT pathway plays an important role in antitumor effects of galangin on CCA cells and we also identify a potential mechanism to explain how galangin exhibits antitumor effects on CCA cells.

Cisplatin plus gemcitabine therapy is the current standard of care for first-line treatment of advanced CCA [7, 24]. Yet, the disease-free survival time is less than 65% after one year and not more than 35% after three years with chemotherapy [6, 25]. Fortunately, results from multiple randomized clinical trials suggested that combined chemotherapy or chemoradiation with adjuvant treatment improves advanced CCA patients' survival [26–30]. For example, patients who received gemcitabine-based adjuvant chemotherapy have significantly improved disease-free survival time by 1.3-fold compared with those patients who did receive gemcitabine only [29]. Hence, select appropriate adjuvant is important for those CCA patients. Flavonoids, a family of naturally occurring polyphenolic compounds represented in multiple plants, are reported to have activity as cancer-preventive agents [31]. Moreover, they are extremely safe and associated with low toxicity, making them excellent candidates for chemotherapy supplemental adjuvant [31]. In this study, using multiple complementary approaches, we demonstrated that galangin, a flavonoid extract from the root of galangal, reduces CCA cell proliferation and induces cell apoptosis (Figure 1). In addition, we also demonstrated that galangin treatment inhibits CCA cell migration and invasion and decreases MMP9 and Vimentin protein expression (Figure 2). These results are consistent with other groups' data that galangin is potent to induce cell apoptosis and inhibit metastasis in other cancer cells [17–19], suggesting stronger antitumor effects of galangin on cancers. Moreover, a previous study found that galangin ameliorates cisplatin-induced nephrotoxicity by attenuating oxidative stress, inflammation, and cell death in a cisplatin-induced acute kidney injury mouse model [32]. Collectively, these data suggest that galangin may serve as a promising adjuvant selected for CCA treatment.

Accumulating evidence demonstrate that miRNAs play an important role in tumor growth and response to chemotherapy through regulating protein expression at the posttranscriptional level [9]. Among these miRNAs, miR-21 is highly overexpressed in CCA tissues from patients and multiple CCA cell lines [10–16]. Patients with high levels of miR-21 present poor prognosis [13]. Subsequent studies confirm that overexpression of miR-21 promotes CCA cell proliferation and metastasis and tumor growth, whereas inhibition of miR-21 exhibits an antitumor phenotype [11, 14–16]. To support, *in vivo* studies demonstrated that overexpression of miR-21 promoted CCA growth and silencing miR-21 expression exhibited an opposite effect using a tumor xenograft mouse model. Furthermore, inhibition of miR-21 sensitizes CCA cells to chemotherapy [12]. These data highlight that miR-21 might be a therapeutic target for CCA treatment. In the current study, we found that galangin potent decreases miR-21 expression in CCA cells (Figure 3(a)). Moreover, the antitumor effects including inhibition of proliferation and metastasis and induction of cell apoptosis of galangin on CCA cells were abrogated by overexpression of miR-21 (Figures 3 and 4), suggesting those galangin-mediated antitumor effects, at least in part, depended on decreasing miR-21 expression.

Enhanced cell proliferation and aberrant metastasis are hallmarks of cancer [33, 34]. Multiple pathways—including PTEN/AKT, MAPK, NF-*κ*B, AMPK, and other signal pathways—are involved in mediating these processes [12, 23, 35, 36]. Among these pathways, the PTEN/AKT pathway is probably the most important one controlling CCA cell survival and apoptosis [12]. Our current study revealed that galangin treatment increases PTEN expression and decreases AKT phosphorylation (Figure 5(a)). To support, CCA cells treated with galangin exhibited a significantly increased expression of Bax and cleaved caspase 3 protein level; both play a key role in regulating cell apoptosis (Figures 1(d) and 1(f)). Yet, those galangin-mediated proapoptotic effects were abrogated by overexpression of miR-21 (Figures 3(e) and 3(f)). These were not surprising; a previous study demonstrated that PETN was a direct target of miR-21 and contributed to miR-21-mediated CCA cell proliferation and resistance to chemotherapy by modulating AKT phosphorylation [12, 14]. AKT is an important kinase involved in controlling cell growth, survival, and apoptosis [37, 38]. A previous study found that the phosphorylation of AKT was increased in CCA tissues [37] and activated AKT promoted CCA cell growth and survival by phosphorylated p27, a cell-cycle inhibitor protein [39]. In accordance with this, we observed that overexpression of miR-21 potent decreases galangin-induced PTEN expression, whereas it increases AKT phosphorylation (Figure 5(a)). Moreover, hyperactivated cell proliferation will result in enhanced tumor migration and invasion [36]. In line with reduced cell proliferation and increased apoptosis in galangin-treated CCA cells, the migration and invasion examined by Matrigel-coated Transwell assessment were also decreased (Figure 2). In contrast, miR-21 overexpression abrogated those galangin-induced phenotypic alterations (Figure 4). Our study indicated that miR-21-mediated PTEN/AKT pathway activation contributes importantly to CCA cell growth and metastasis, which can be inhibited by galangin treatment.

## 5. Conclusion

Taken together, those data demonstrate that galangin inhibits proliferation and metastasis and promotes apoptosis in both HCCC9810 and TFK-1 cells in a miR-21-dependent manner (Figure 5(b)). Our findings provide new evidence that miR-21 can be a therapeutic target for CCA and suggest that galangin may provide a novel potential therapeutic adjuvant from natural products for the treatment of CCA.

## Data Availability

All data is available in the main text.
